# Safety, immunogenicity and protective effectiveness of heterologous boost with a recombinant COVID-19 vaccine (Sf9 cells) in adult recipients of inactivated vaccines

**DOI:** 10.1038/s41392-024-01751-1

**Published:** 2024-02-14

**Authors:** Wenxin Luo, Jiadi Gan, Zhu Luo, Shuangqing Li, Zhoufeng Wang, Jiaxuan Wu, Huohuo Zhang, Jinghong Xian, Ruixin Cheng, Xiumei Tang, Yi Liu, Ling Yang, Qianqian Mou, Xue Zhang, Yi Chen, Weiwen Wang, Yantong Wang, Lin Bai, Xuan Wei, Rui Zhang, Lan Yang, Yaxin Chen, Li Yang, Yalun Li, Dan Liu, Weimin Li, Lei Chen

**Affiliations:** 1 Department of Pulmonary and Critical Care Medicine, West China Hospital, Sichuan University, Chengdu, China; 2 Institute of Respiratory Health, Frontiers Science Center for Disease-related Molecular Network, West China Hospital, Sichuan University, Chengdu, China; 3 Precision Medicine Center, Precision Medicine Key Laboratory of Sichuan Province, West China Hospital, Sichuan University, Chengdu, China; 4 State Key Laboratory of Respiratory Health and Multimorbidity, West China Hospital, Chengdu, China; 5 Institute of Respiratory Health and Multimorbidity, West China Hospital, Sichuan University, Chengdu, China; 6 Clinical Trial Center, West China Hospital, Sichuan University, Chengdu, China; 7 General Practice Medical Center, West China Hospital, Sichuan University, Chengdu, China; 8 Fangcao Community Health Service Center of Chengdu High-tech Zone, Chengdu, China; 9 The Research Units of West China, Chinese Academy of Medical Sciences, West China Hospital, Chengdu, China; 10 West China School of Nursing, Sichuan University, Chengdu, China; 11 Department of Infection Control, West China Hospital, Sichuan University, Chengdu, China; 12 Department of Pathology, West China Hospital, Sichuan University, Chengdu, China; 13 Department of Respiratory and Critical Care Medicine, the First Affiliated Hospital of Chongqing Medical University, Chongqing, China; 14 Department of Neurology, West China Hospital, Sichuan University, Chengdu, China

**Keywords:** Health policy, Clinical trial design, Health care

## Abstract

Vaccines have proven effective in protecting populations against COVID-19, including the recombinant COVID-19 vaccine (Sf9 cells), the first approved recombinant protein vaccine in China. In this positive-controlled trial with 85 adult participants (Sf9 cells group: *n* = 44; CoronaVac group: *n* = 41), we evaluated the safety, immunogenicity, and protective effectiveness of a heterologous boost with the Sf9 cells vaccine in adults who had been vaccinated with the inactivated vaccine, and found a post-booster adverse events rate of 20.45% in the Sf9 cells group and 31.71% in the CoronaVac group (*p* = 0.279), within 28 days after booster injection. Neither group reported any severe adverse events. Following the Sf9 cells vaccine booster, the geometric mean titer (GMT) of binding antibodies to the receptor-binding domain of prototype SARS-CoV-2 on day 28 post-booster was significantly higher than that induced by the CoronaVac vaccine booster (100,683.37 vs. 9,451.69, *p* < 0.001). In the Sf9 cells group, GMTs of neutralizing antibodies against pseudo SARS-CoV-2 viruses (prototype and diverse variants of concern [VOCs]) increased by 22.23–75.93 folds from baseline to day 28 post-booster, while the CoronaVac group showed increases of only 3.29–10.70 folds. Similarly, neutralizing antibodies against live SARS-CoV-2 viruses (prototype and diverse VOCs) increased by 68.18–192.67 folds on day 14 post-booster compared with the baseline level, significantly greater than the CoronaVac group (19.67–37.67 folds). A more robust Th1 cellular response was observed with the Sf9 cells booster on day 14 post-booster (mean IFN-γ+ spot-forming cells per 2 × 10^5^ peripheral blood mononuclear cells: 26.66 vs. 13.59). Protective effectiveness against symptomatic COVID-19 was approximately twice as high in the Sf9 cells group compared to the CoronaVac group (68.18% vs. 36.59%, *p* = 0.004). Our study findings support the high protective effectiveness of heterologous boosting with the recombinant COVID-19 vaccine (Sf9 cells) against symptomatic COVID-19 of diverse SARS-CoV-2 variants of concern, while causing no apparent safety concerns.

## Introduction

Since its emergence in 2019, severe acute respiratory syndrome coronavirus 2 (SARS-CoV-2) infection has been associated with more than 769.81 million cases and resulted in more than 6.96 million deaths worldwide as of Aug 18, 2023.^1^ Vaccination against SARS-CoV-2 has significantly reduced the burden of corona virus disease 2019 (COVID-19). Globally, 13.50 billion doses of COVID-19 vaccines had been administered by Aug 18 2023.^1^ The World Health Organization (WHO) reported that more than 350 COVID-19 vaccines were in preclinical or clinical development, with 34 COVID-19 vaccines approved for marketing worldwide.^2^ These vaccines are mainly of four types, namely inactivated virus, messenger RNA (mRNA), adenovirus vector-based, and adjuvanted protein vaccines.^3^ The inactivated vaccines (e.g. BBIBP-CorV by Sinopharm and CoronaVac by Sinovac), the mRNA vaccines (e.g. mRNA-1273 by Moderna and BNT162b2 by Pfizer-BioNTech), the adenovirus vaccines (e.g. Ad26-S.PP by Johnson &Johnson’s and ChAdOx1 by AstraZeneca), and the adjuvanted protein vaccine (e.g. SCB-2019 by Clover Biopharmceuticals and CoVLP+AS03 by Medicago) have been widely used.^4–10^

Variants of concern (VOCs) have become a contributing factor to the rising infection rates of SARS-CoV-2 within the vaccinated population.^11^But emerging VOCs and breakthrough infections highlight the need for COVID-19 vaccines with enhanced protective efficacy and prolonged duration of protection^12,13^ Based on the prototype SARS-CoV-2 (HB-01) a recombinant COVID-19 vaccine (Sf9 cells) was designed and produced, also referred to as Sf9 cells recombinant vaccine in this study, which is a protein subunit vaccine comprised of the antigen of the tandem receptor-binding domain (RBD) of the spike protein.^14^ Phase I and II clinical studies have demonstrated promising anti-virus activity and tolerability of the Sf9 cells recombinant vaccine.^15^ Binding and neutralizing antibody test revealed that the Sf9 cells recombinant vaccine had brilliant immunogenicity. The Sf9 cells recombinant vaccine also triggered a CD4+ type 1 helper T (Th1) cell response and robust production of interferon-γ (IFN-γ). The ability to stimulate both strong humoral and cellular antiviral activities of the Sf9 cells recombinant vaccine makes it a promising vaccine. The Sf9 cells recombinant vaccine was first granted emergency use authorization in China on Dec 5, 2022. It became the first recombinant protein vaccine against SARS-CoV-2 approved in China and has since received authorization in multiple countries globally.

Combining vaccines of different technological platforms during the primary and booster phases refers to the strategy of administering a heterologous booster vaccine after one-dose priming, two-dose homologous priming, or three-dose homologous priming. Compared with homologous inactivated-only vaccine schedules, “heterologous boosting” using vaccines of different technological platforms enhances the protection of inactivated SARS-CoV-2 vaccines.^16^ Evidence indicated that two inactivated SARS-CoV-2 vaccine (CoronaVac), also referred to as CoronaVac inactivated vaccine here, doses plus a third homologous booster one of inactivated CoronaVac could increase neutralizing antibody titers against SARS-CoV-2 viruses (e.g. Alpha and Delta variants), but with low neutralization responses against the Omicron variant.^17^ Using heterologous boosters (such as mRNA vaccine CS-2034 or adenovirus vaccine Ad5-nCoV) after two inactivated CoronaVac vaccine doses induced significant improvements in immune responses and enhanced protective effectiveness, compared to a third dose of homologous CoronaVac vaccine.^17–19^ However, we still lack safety and immunogenicity data comparing the heterologous adjuvanted protein vaccine as the fourth dose with homologous vaccination schedules.

To assess the protective effectiveness of a fourth heterologous booster vaccination with an adjuvanted protein vaccine and provide evidence for the development of sound booster strategies, we designed a positive-control clinical trial. It aimed to determine the safety, immunogenicity, and protective effectiveness of the recombinant COVID-19 vaccine (Sf9 cells) as a heterologous booster for healthy adults having received three inactivated vaccines at the latest six months before.

## Result

### Participants

**Fig. 1:**
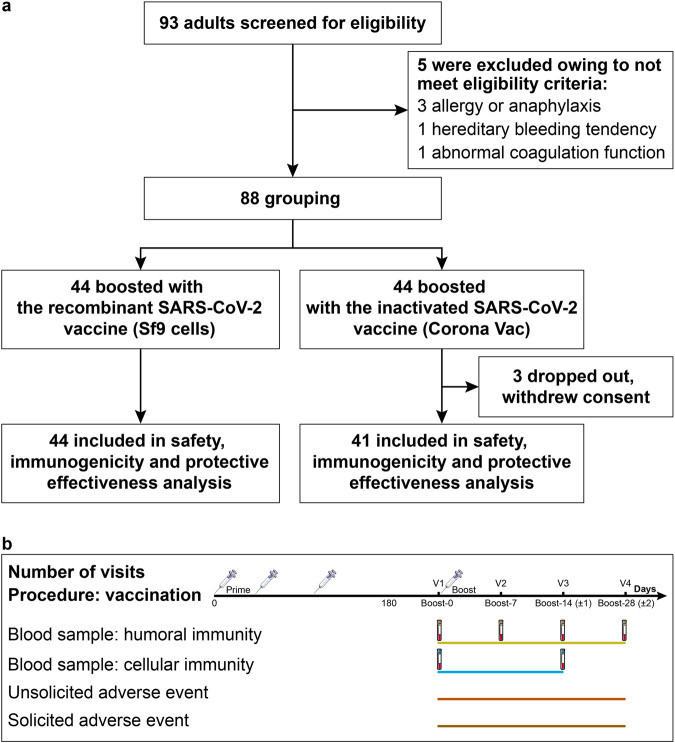
Study design and procedure. **a** Flowchart of participant enrollment. **b** Immunization schedule and blood sample collection in Sf9 cells group and CoronaVac group. Blood samples were collected at Baseline (day at the booster, before the boost), Day 7 (7 days after the boost), Day 14 (14 days after the boost), and Day 28 (28 days after the boost). V visit

We recruited and screened 93 participants and included 88 to be assigned to receive injection of Sf9 cells recombinant vaccine (*n* = 44) or CoronaVac inactivated vaccine (*n* = 44) until Nov 18, 2022 (Fig. 1a). Three participants later dropped out of the study by withdrawing consent, so a total of 85 nucleic acid-negative participants (Sf9 cells group: *n* = 44; CoronaVac group: *n* = 41) were eligible for safety, immunogenicity, and protective effectiveness analysis. All participants visited within 28 days as planned for safety and immunogenicity evaluation. The protective effectiveness follow-up was from day 15 after receiving the booster vaccination to Jan 10, 2023. Blood specimens were collected on Days 0 (Baseline), 7, 14, and 28 after booster vaccination, for measuring specific binding antibodies to RBD and neutralizing antibodies against live and pseudo viruses of SARS-CoV-2. Simultaneously, we also isolated peripheral blood mononuclear cells (PBMCs) at Baseline and on Day 14 to evaluate the specific Th1 immune response to SARS-CoV-2 (Fig. 1b).

The Sf9 cells and the CoronaVac groups were comparable in respects of baseline characteristics of age, sex, height, weight, body-mass index (BMI), and the time interval from the third vaccination to the fourth booster (*p* > 0.05 for all; Table 1). Six participants had coexisting conditions, including one (diabetes) of the Sf9 cells group and five (two with hypertension, two chronically infected with hepatitis B virus and one with bilateral renal calculus) from CoronaVac group.

**Table 1:** Baseline characteristics of the participants stratified by type of boost vaccination

Characteristic	All (*n* = 85)	Heterologous boost with Sf9 cells vaccine (*n* = 44)	Homologous boost with CoronaVac vaccine (*n* = 41)	*p* value
**Age, years**				0.363
Median (Min, Max)	49 (23, 70)	49 (25, 67)	46 (23, 70)	
**Age subgroup,** ***n*** **(%)**				0.039
18–59 years	67 (78.80)	35 (79.50)	32 (78.05)	
≥60 years	18 (21.20)	9 (20.40)	9 (21.95)	
**Sex,** ***n*** **(%)**				0.881
Male	40 (47.10)	22 (50.0)	18 (43.90)	
Female	45 (52.90)	22 (50.0)	23 (56.10)	
**Height, cm**				0.838
Median (Min,Max)	164.0 (136.5, 180.0)	164.0 (136.5, 180.0)	164.0 (149.0, 179.5)	
**Weight, kg**				0.996
Median (Min Max)	61.4 (42.0, 98.5)	61.8 (42.0, 82.0)	61.1 (48.5, 98.5)	
**BMI, kg/m**^**2**^				0.883
Median (Min, Max)	23.7 (17.5, 33.3)	23.7 (17.5, 33.0)	23.7 (18.3, 33.3)	
Preexisting co-morbidities*, ***n*** **(%)**				0.043
Yes	6 (7.06)	1 (2.27)	5 (12.19)	
No	79 (92.94)	43 (97.73)	36 (87.80)	
**Time between third vaccination and the fourth booster doses in the present study, days**				0.178
Mean (±SD)	286.09 ( ± 44.57)	277.84 ( ± 48.19)	294.95( ± 38.97)	
Median (Min, Max)	283 (220, 351)	273 (220, 351)	313 (222, 336)	

^*^Two participants had hypertension, two were chronically infected with hepatitis B virus, one had bilateral renal calculus, one had diabetes. *Min* minimum; *Max* maximum, *SD* standard deviation, *BMI* body mass index

### Safety

Overall, after the booster Sf9 cells recombinant vaccine or CoronaVac inactivated vaccine, 9 participants (20.45%) and 13 participants (31.71%) respectively reported at least one adverse event (AE), but without statistically significant difference (*p* = 0.279) (Table 2). All AEs were mild (Grade 1) or moderate (Grade 2); no Grade 3 or higher AEs were reported; nobody dropped out due to AEs. The most common AE was solicited pain at injection-site within 7 days in both the Sf9 cells group (5/44, 11.36%) and the CoronaVac group (6/41, 14.63%), but with no significant difference between two groups (*p* = 0.654). Fatigue was the most reported solicited systemic AE within 7 days in both the Sf9 cells group (1/44, 2.27%) and the CoronaVac group (3/41, 7.32%) but did not show significant difference between them (*p* = 0.272). Unsolicited AEs within 28 days were significantly less in the Sf9 cells group than the CoronaVac group (4.55% vs. 21.95%, *p* = 0.017).

**Table 2:** Comparison of incidence of adverse events within 28 days after different types of boost vaccination

Adverse event	All (*n* = 85)	Heterologous boost with Sf9 cells vaccine (*n* = 44)	Homologous boost with CoronaVac vaccine (*n* = 41)	*p* value
**Any adverse event,** ***n*** **(%)**	22 (25.88)	9 (20.45)	13 (31.71)	0.279
Grade 1	17 (20.00)	8 (18.18)	9 (21.95)	0.664
Grade 2	5 (5.88)	1 (2.27)	4 (9.76)	0.143
Grade 3 or worse	0 (0)	0 (0)	0 (0)	—
**Solicited adverse event within 7 days,** ***n*** **(%)**	17 (20.00)	7 (15.91)	10 (24.39)	0.329
**Solicited adverse event at the injection site within 7 days,** ***n*** **(%)**	11(12.94)	5 (11.36)	6 (14.63)	0.654
Pain	11 (12.94)	5 (11.36)	6 (14.63)	0.654
Swelling	0 (0)	0 (0)	0 (0)	—
Induration	0 (0)	0 (0)	0 (0)	—
Erythema	0 (0)	0 (0)	0 (0)	—
**Solicited systemic adverse event within 7 days,** ***n*** **(%)**	8 (9.41)	3 (6.82)	5 (12.20)	0.453
Fatigue	4 (4.70)	1 (2.27)	3 (7.32)	0.272
Headache	3 (3.53)	1 (2.27)	2 (4.88)	0.515
Nausea	1 (1.20)	0 (0)	1 (2.44)	0.297
Arthralgia	1 (1.20)	1 (2.27)	0 (0)	0.332
Fever	0 (0)	0 (0)	0 (0)	—
Vomiting	0 (0)	0 (0)	0 (0)	—
Myalgia	0 (0)	0 (0)	0 (0)	—
**Unsolicited adverse event within 28 days,** ***n*** **(%)**	11 (12.94)	2 (4.55)	9 (21.95)	0.017

### Humoral immunogenicity

The Sf9 cells recombinant vaccine elicited stronger specific binding antibody response to RBD compared to the CoronaVac inactivated vaccine (Figure S1 and Table S1). Following the booster vaccination, a significant enhancement in the binding antibody to RBD from Day 0 to Day 28 was found in recipients of the Sf9 cells recombinant vaccine. The geometric mean titers (GMTs) for the binding antibody to RBD in the Sf9 cells group was 3052.29 (95% CI 2366.06–3937.54) at day 0, 25199.87 (15528.41–40894.95) at day 7, 100799.50 (66053.54–153822.76) at day 14, and 100683.37 (68820.24–147298.84) at day 28, respectively. In comparison, the GMTs for binding antibody to RBD in the CoronaVac group was 3986.56 (95% CI 2846.18–5583.87) at day 0, 6884.42 (5131.20-9236.67) at day 7, 10993.35 (8282.00–14592.34) at day 14, and 9451.69 (6565.94–13605.73) at day 28 post-booster immunization, respectively. Notably, the elevation in the Sf9 cells group was more significant than that observed in the CoronaVac cells group.

**Fig. 2:**
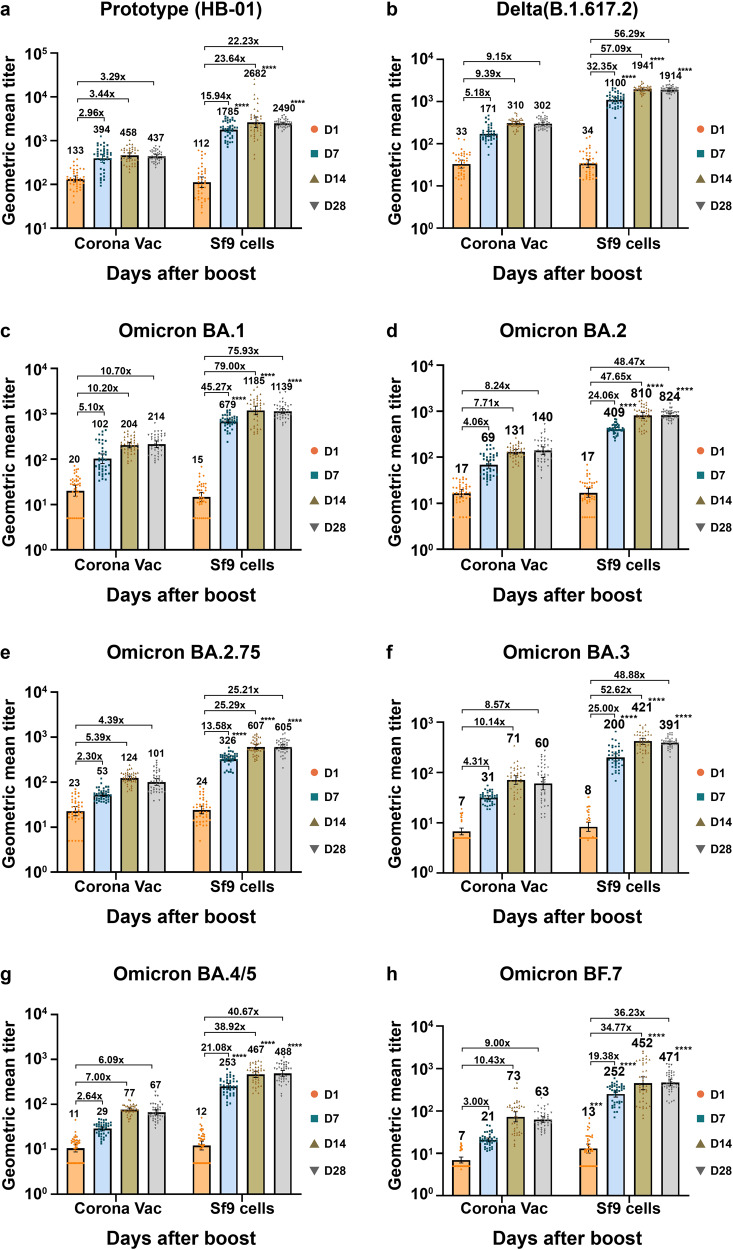
Geometric mean titers of neutralizing antibodies against different variants of pseudo SARS-CoV-2 virus before and after the booster vaccination. **a**–**h**. Neutralizing antibodies against different SARS-CoV-2 variants of pseudo virus. Bars and associated numbers represent geometric means, and boxes 95% CIs. **** *p* < 0.0001 (Unpaired two-sided Student’s t test). CI Confidence interval

In recipients of heterologous Sf9 cells recombinant vaccine we found a significantly higher neutralizing antibody response against pseudo SARS-CoV-2 virus (both prototype [HB-01] and VOCs) compared to those of homologous CornonaVac inactivated vaccine (Fig. 2 and Table S2). The geometric mean fold increase (GMFI) of neutralizing antibodies against SARS-CoV-2 pseudo viruses (prototype [HB-01], Delta [B.1.617.2], and Omicron [BA.1; BA.2; BA.2.75; BA.3; BA.4/5; BF.7]) at day 28 post-booster vaccination ranged from 22.23- to 75.93-fold for participants receiving the Sf9 cells recombinant vaccine. In contrast, for participants receiving inactivated CoronaVac vaccine the GMFI of neutralizing antibodies against SARS-CoV-2 pseudo virus at day 28 post-booster vaccination ranged from 3.29- to 10.70-fold. In the Sf9 cells group, the neutralizing antibodies against SARS-CoV-2 pseudo virus (Omicron [BA.1]) increased the most, with a GMT of 14.72 (11.63–18.63) at Day 0, 679.36 (609.76–756.90) at Day 7, 1185.27 (956.56–1468.67) at Day 14, and 1138.72 (1005.77–1289.25) at Day 28. Those against pseudo SARS-CoV-2 virus (prototype [HB-01]) increased the least, with a GMT of 112.09 (95%CI 85.14–147.57) at Day 0, 1785.27 (1547.64–2059.39) at Day 7, 2628.47 (1991.91–3468.45) at Day 14, and 2490.09 (2340.48–2649.27) at Day 28. Similarly, for the CoronaVac group, the neutralizing antibodies against pseudo virus (Omicron [BA.1]) increased the most, with a GMT of 20.25 (15.26–26.87) at day 0, 102.46 (80.65-130.16) at day 7, 204.49 (178.75-233.94) at day 14, and 214.34 (182.00–252.43) at day 28. Conversely, the neutralizing antibodies against pseudo SARS-CoV-2 virus (prototype [HB-01]) increased the least, with a GMT of 132.77 (95%CI 113.39–155.46) at day 0, 394.36 (320.71–484.94) at day 7, 458.01 (400.92–523.24) at day 14, and 436.95 (399.71–477.66) at day 28. Notably, the GMT for neutralizing antibodies against SARS-CoV-2 pseudo viruses of all gene types stabilized after around Day 14 post booster vaccinations of both Sf9 cells and CoronaVac vaccines. The GMTs remained almost the same at Day 14 and Day 28 poster booster vaccination. We observed significantly higher GMTs against prototype HB-01 and Omicron variants post Sf9 cells vaccination than after CoronaVac vaccination.

**Fig. 3:**
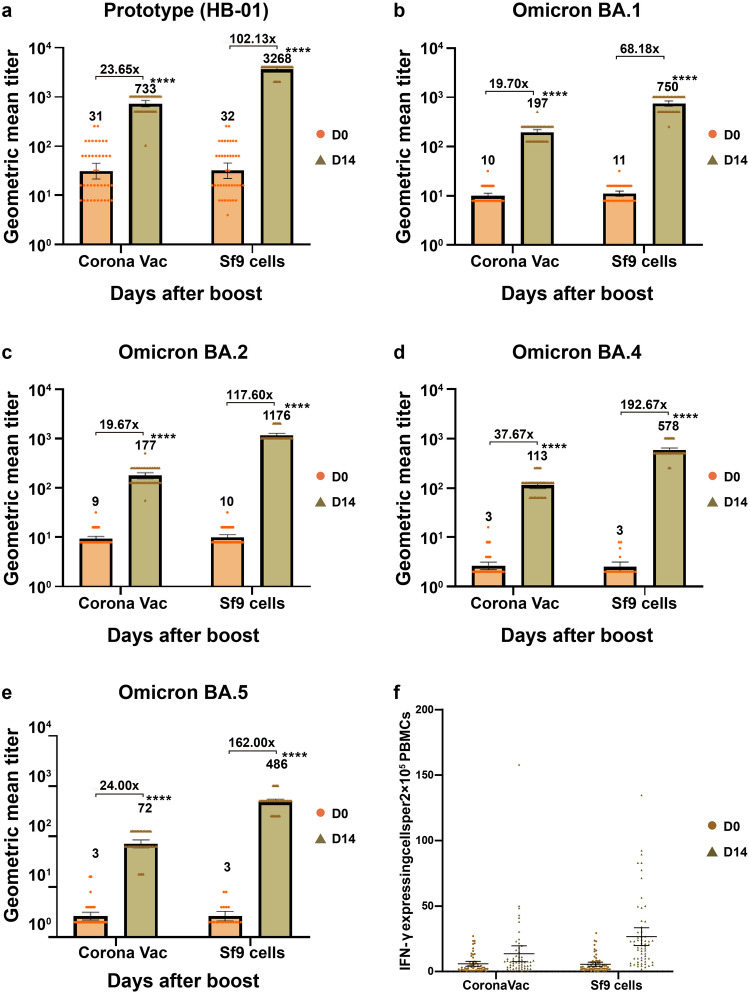
Geometric mean titers of neutralizing antibodies against different SARS-CoV-2 variants of live virus before and after the booster vaccination and specific Th1 cell reactivity on day 14 after booster vaccination. **a**–**e** Neutralizing antibodies against different SARS-CoV-2 variants of live virus. Bars and associated numbers represent geometric means, and boxes 95% Cl. CI Confidence interval. **f** Spot-forming cells with secretion of IFN-γ cytokines per 2 × 10^5^ PBMCs measured by ELISpot. *IFN-γ* Interferon-γ, PBMC peripheral blood mononuclear cell, *ELISpot* Enzyme-linked immunospot. **** *p* < 0.0001. CI Confidence interval

Compared to the CoronaVac group, the Sf9 cells group exhibited higher neutralizing antibody response against live SARS-CoV-2 viruses of both prototype [HB-01] and VOCs (Fig. 3a–e and Table S2). The GMFI of neutralizing antibodies against live SARS-CoV-2 virus (prototype [HB-01], Omicron [BA.1; BA.2; BA.4and BA.5]) at Day 14 post-booster ranged from 68.18- to 192.67-folds for participants receiving the Sf9 cells recombinant vaccine; whereas for participants receiving the CoronaVac inactivated vaccine the GMFI of neutralizing antibodies against live SARS-CoV-2 virus at Day 14 post-booster ranged from 19.67- to 37.67-folds. For the Sf9 cells recombinant group, the neutralizing antibodies against live Omicron [BA.4] virus increased the most, with a GMT of 2.51 (95%CI 2.00–3.15) at day 0 and 578.03 (517.31–645.88) at day 14; whereas those against Omicron [BA.1] live virus increased the least, with a GMT of 11.12 (9.83–12.57) at day 0 and 749.61 (663.22–847.26) at day 14. For the CoronaVac group, the neutralizing antibodies against Omicron [BA.4] live SARS-CoV-2 virus increased the most, with a GMT of 2.64 (2.22–3.14) at day 0 and 113.38 (98.47–130.54) at day 14; whereas those against Omicron [BA.2] live virus increased the least, with a GMT of 9.35 (8.41–10.40) at day 0 and 177.32 (154.84–203.05) at day 14.

### Cellular immunogenicity

The heterologous Sf9 cells recombinant vaccine elicited a more robust Th1-mediated IFN-γ production compared to the homologous CoronaVac inactivated vaccine. Fourteen days after booster vaccination, the mean IFN-γ+ spot-forming cells were 26.66 per 2 × 10^5^ PBMCs (95% CI, 33.46–49.87) in the Sf9 cells group and 13.59 per 2 × 10^5^ PBMCs (95% CI, 7.49–19.69) in the CoronaVac group (*p* < 0.05, Student’s *t* test; Fig. 3f). In the Sf9 cells group, 91% of participants exhibited positive IFN-γ responses, whereas in the CoronaVac group, the percentage was 75%, with significant difference between the groups (*p* < 0.05, Chi-square test).

### Protective effectiveness against symptomatic COVID-19

Among the 85 participants included in this trial, 31.82% (14/44) in the Sf9 cells group and 63.41% (26 /41) in the CoronaVac group were confirmed symptomatic COVID-19 from 15 days after booster vaccination through January 10, 2023. The protective effectiveness of Sf9 cells recombinant vaccine against symptomatic COVID-19 was nearly twice that of CoronaVac inactivated vaccine (68.18% vs. 36.59%, *p* = 0.004, Chi-square test; Table S3). When considering participants in different age subgroups, the Sf9 cells recombinant vaccine exhibited better protective effectiveness against symptomatic disease than CoronaVac inactivated vaccine in participants aged 18–59 (72.73% vs. 39.02%, *p* = 0.002, Chi-square test); however, no significant difference was found in participants aged 60 years or older (95.45% vs. 97.60%, *p* > 0.05, Chi-square test). Fewer patients in the Sf9 cells group reported three or more symptoms compared to the CoronaVac group (6.82% vs. 24.39%, *p* = 0.025, Chi-square test).

## Discussion

Our study is the first one to provide clinical evidence supporting a heterologous booster immunization with Sf9 recombinant vaccine in adult recipients of inactivated vaccine. The safety profile of Sf9 cells recombinant vaccine as a booster dose was determined to be comparable to that of a booster dose with CoronaVac inactivated vaccine following a three-dose priming with CoronaVac vaccine. When AEs did occur, they were of low grades (Grade 1 and Grade 2), with the most often reported AE as solicited injection-site pain within 7 days. One possible explanation could be attributed to the choice of Sf9 cells recombinant vaccine as the test vaccine. Using Baculovirus Expression Vector System (BEVS) for producing Sf9 cells recombinant vaccine ensures great safety for mammals, as the majority of the recombinant protein remains primarily contained within the BEVS.^15^ Our small-scale trial offers evidence that the Sf9 cells recombinant vaccine can be a safe heterologous booster for individuals having had three doses of inactivated COVID-19 vaccination.

Our research aligns with prior studies indicating that heterologous boosters against SARS-CoV-2 are not only well-tolerated but also more effective in eliciting protective immune responses compared to homologous boosters.^20–25^ The same may also apply to vaccines against other viruses.^26^ The effectiveness of COVID-19 vaccination tends to decrease as individuals age and in the presence of underlying health conditions^27^ Conducting head-to-head protective effectiveness trials becomes challenging due to limitations such as insufficient trial participants, challenges in vaccine promotion efforts, and the presence of inherent immunity within the population. This trial compared the tested vaccine (Sf9 cells recombinant vaccine) with the positive control vaccines (CoronaVac inactivated vaccine) to assess protective effectiveness in balanced groups during the same period. Vaccine protective effectiveness, distinct from vaccine efficacy (1 minus the relative risk ratio), means decreased risks of SARS-CoV-2 infection or COVID-19 disease observed among the participants. Our trial showed that as a booster the heterologous Sf9 cells recombinant vaccine provided nearly double protection effectiveness against symptomatic COVID-19 compared with the homologous inactivated CoronaVac vaccine (68.20% vs. 36.60%) in the subsequent days of follow-up, aligning with increased immunogenicity against SARS-CoV-2 viruses.

In our analysis, boosters of both Sf9 cells recombinant vaccine and CoronaVac inactivated vaccine significantly elevated humoral and cellular immune responses. This suggests that either booster is effective for individuals previously vaccinated with inactivated SARS-CoV-2 vaccine. However, a booster dose of Sf9 cells recombinant vaccine elicited a superior humoral immune response, eg. higher titers of neutralizing antibodies against various subtypes of SARS-CoV-2 pseudo-virus, especially Delta (B.1.617.2) and Omicron BA.1 variants, by as early as one week after the booster vaccination. Additionally, neutralizing antibodies against the pseudo-virus Omicron BA.1 increased from baseline by approximately 79.00 times two weeks after the heterologous booster with Sf9 cells recombinant vaccine and maintained a consistently high value of 75.93 times after an additional two weeks. On the contrary, at the corresponding time points following the homologous booster with CoronaVac inactivated vaccine, GMT was approximately 10.70 times at 2 weeks and only 10.70 times at 4 weeks. Similar trends were observed for other viral subtypes. Thus, we reason that compared to the homologous boost, the heterologous booster regimen could trigger stronger and faster antigen-specific immune responses. This can be attributable to activation of naive B cells, followed by subsequent plasma cell activation, leading to a significant enhancement in humoral immune responses.^28^

In addition to humoral immune responses, equally important are T-cell immune responses elicited by the vaccine, including Th1 mediated IFN-γ production. An Sf9 cells booster dose resulted in a swift elevation of T-cell responses, suggesting the prompt recall of pre-existing immunity induced by the initial CoronaVac vaccine regimen. Moreover, in this trial, Th1 cells demonstrated the ability to recognize various VOCs, consistent with observations from previous reports. This addresses concerns raised by the public.^29^ Such T cell responses can complement antibody-mediated protection against SARS-CoV-2.^30,31^ Hence, this study suggests that activation of Th1 cells plays a crucial role in response to declining antibody levels and emerging SARS-CoV-2 VOCs.

Since this study had a small sample size and one single ethnicity, its results are up to validation and extension by future studies, which can also evaluate long-term safety and immunogenicity. Additionally, although we only conducted a brief one-month follow-up after the booster, it coincided with the second wave of the pandemic in China. Continuous monitoring of protective efficacy over the six months following the last booster would be more valuable because, as reported, the protective effectiveness of boosting may decline within two months after the fourth dose.^32^ Despite these limitations, our trial provides evidence for safety and protective effectiveness of heterologous booster vaccination.

## Materials and methods

### Study design and participants

We conducted a single-center, double-blind, positive-control clinical trial to assess the safety, immunogenicity, as well as protective effectiveness of heterologous booster immunization using an adjuvanted protein vaccine, the Sf9 cells recombinant vaccine. The trial received approval from the Institutional Review Board of West China Hospital, Sichuan University (No. 2022-1226), and was registered at the Chinese Clinical Trial Register (ChiCTR2200062403). The study protocol adhered strictly to the guidelines of the Declaration of Helsinki and Good Clinical Practice.

Participants were enrolled in the trial in Chengdu, Sichuan Province, China, up to November 18, 2022. Eligible participants were healthy adults (≥18 years) who had received three doses of inactivated COVID-19 vaccine at the latest 6 months prior to the screening visit. Exclusion criteria included positive nucleic acid test result for SARS-CoV-2 at screening, axillary temperature exceeding 37.3 °C, any previous COVID-19 or infection of SARS-CoV-2 or other coronaviruses (eg., MERS-CoV or SARS-CoV). Also excluded were individuals allergic to any vaccine component and women having positive urine pregnancy test results. All criteria for inclusion and exclusion are listed in Table S4. Written informed consent was signed by every participant upon enrollment.

### Grouping and vaccines

Enrolled participants were sequentially assigned, based on the order of enrollment, to receive either a heterologous booster of Sf9 cells recombinant vaccine or a homologous fourth dose of CoronaVac inactivated vaccine. Only investigators knew details about grouping. Participants and personnel responsible for testing biological samples were blinded until all data had been collected. The Sf9 cells recombinant vaccine is manufactured by WestVac BioPharma Co., Ltd., and has been granted conditional licenses or emergency use authorization against COVID-19 in China and other countries. The baculovirus-vectored vaccine adjuvanted with aluminum hydroxide expresses the SARS-CoV-2 spike protein receptor-binding domain in Sf9 cells. The positive control vaccine, the CoronaVac inactivated vaccine, is developed by Beijing Sinovac Research & Development Co., Ltd., and has been proven for safety and effectiveness in the COVID-19 pandemic within and outside China.^33,34^

### Procedures

After the screening process, all enrolled participants were administered a single booster shot of either Sf9 cells recombinant vaccine or CoronaVac inactivated vaccine. All recipients were monitored for 30 minutes after vaccination for any vaccine-related immediate adverse reactions. Additionally, participants were required to maintain a daily record of solicited AEs for 7 days and unsolicited AEs for 28 days after the booster shot. Any reported serious AEs were monitored for fully 6 months post vaccination. The severity of adverse events was graded in four levels: Grade 1 (mild), Grade 2 (moderate), Grade 3 (severe), or Grade 4 (life-threatening), according to the toxicity grading criteria of the National Medical Products Administration, China (NMPA).^35^

Blood samples were collected from all participants at baseline (prior to booster vaccination) and on days 7, 14, and 28 post booster vaccination. These samples were then processed to isolate PBMCs and serum for immunogenicity assays. The assessment of humoral immunogenicity included analyzing binding antibody responses (IgG) specific to the RBD and measuring neutralizing antibody activities against pseudo and live SARS-CoV-2 viruses. To measure binding antibodies specific to the prototype RBD, an enzyme-linked immunospot assay (ELISA) kit was utilized. The neutralizing antibody titer was determined with both live-virus and pseudo-virus neutralization assays, as previously described.^15^ To assess cross-neutralizing activities, the neutralization assay incorporated prototype SARS-CoV-2 viruses and VOCs, including Delta and Omicron strains. The SARS-CoV-2-specific T-cell response was determined by IFN-γ ELISpot assay in PBMCs, as previously described.^15^ All the 44 Sf9 cells group participants and the 41 CoronaVac group ones were included in analysis of specific binding antibody response to RBD. Neutralizing antibody responses and specific T cell immune responses were measured in 40 participants from each group.

### Outcomes

The primary endpoint of safety for this study was determined as the occurrence of solicited adverse events within 7 days post booster shot. The primary endpoints of immunogenicity included specific binding antibody responses to the RBD and the GMTs of neutralizing antibodies against pseudo SARS-CoV-2 viruses at days 7, 14, and 28 after the booster shot. The secondary endpoint of safety was determined as the incidence of unsolicited adverse events within 28 days after the booster vaccination and serious adverse events (SAEs) up to 6 months post booster. The secondary endpoint of immunogenicity for this study was determined as GMTs of neutralizing antibodies against live SARS-CoV-2 viruses at 14 days after booster shot. Exploratory outcomes involved specific Th1 cell immune responses against SARS-CoV-2 determined by IFN-γ ELISpot assay in PBMCs at Day 14 post booster.

The new COVID-19 pandemic emerged in China after December 7, 2022, when the country fully lifted its epidemic prevention and control policies.^19,36^ Consequently, we had the opportunity to assess and compare the protective effectiveness of a fourth booster of the Sf9 cells recombinant vaccine and CoronaVac inactivated vaccine in participants of this trial. The protective effectiveness was indicated as decreased risks of symptomatic COVID-19 (with onset time no earlier than 15 days post booster). COVID-19 disease was diagnosed according to the case definition determined by the WHO (updated July 10, 2023).^37^ Symptomatic COVID-19 was diagnosed based on symptoms and epidemic contact history (PCR or antigen detected positively, partly available) with onset at least 15 days after receiving the Sf9 cells recombinant vaccine or CoronaVac inactivated vaccine boost. The diagnostic criteria are listed in Table S5.

### Statistical analysis

Statistical analyses were performed with GraphPad Prism 9.1.2 (GraphPad Software, La Jolla, CA, USA) and RStudio 4.0.5 (RStudio Software, Boston, MA, USA). All tests applied were two-tailed. Statistical results associated with *p* < 0.05 were considered significant. Categorical variables were demonstrated as counts and percentages; the statistical significance of differences between two groups was determined by either the Chi-square test or Fisher’s exact test, depending on appropriateness. Continuous variables were analyzed for significance using the Student’s t-test (normally distributed data) and the Wilcoxon rank-sum test (non-normally distributed data). Normality of data was determined with the Shapiro-Wilk test.

### Supplementary information

Supplementary Figure 1

Supplementary Materials

## Data Availability

Data involved in this study will be made available upon request to the corresponding authors, including anonymized participant data, and additional documents like the study protocol, statistical analyses and informed consents.
