# Supplementary figures and images for: Safety, immunogenicity and protective effectiveness of heterologous boost with a recombinant COVID-19 vaccine (Sf9 cells) in adult recipients of inactivated vaccines
Source: Signal Transduct Target Ther. 2024 Feb 14;9:41. doi: 10.1038/s41392-024-01751-1 (PMC10866951; doi:10.1038/s41392-024-01751-1)

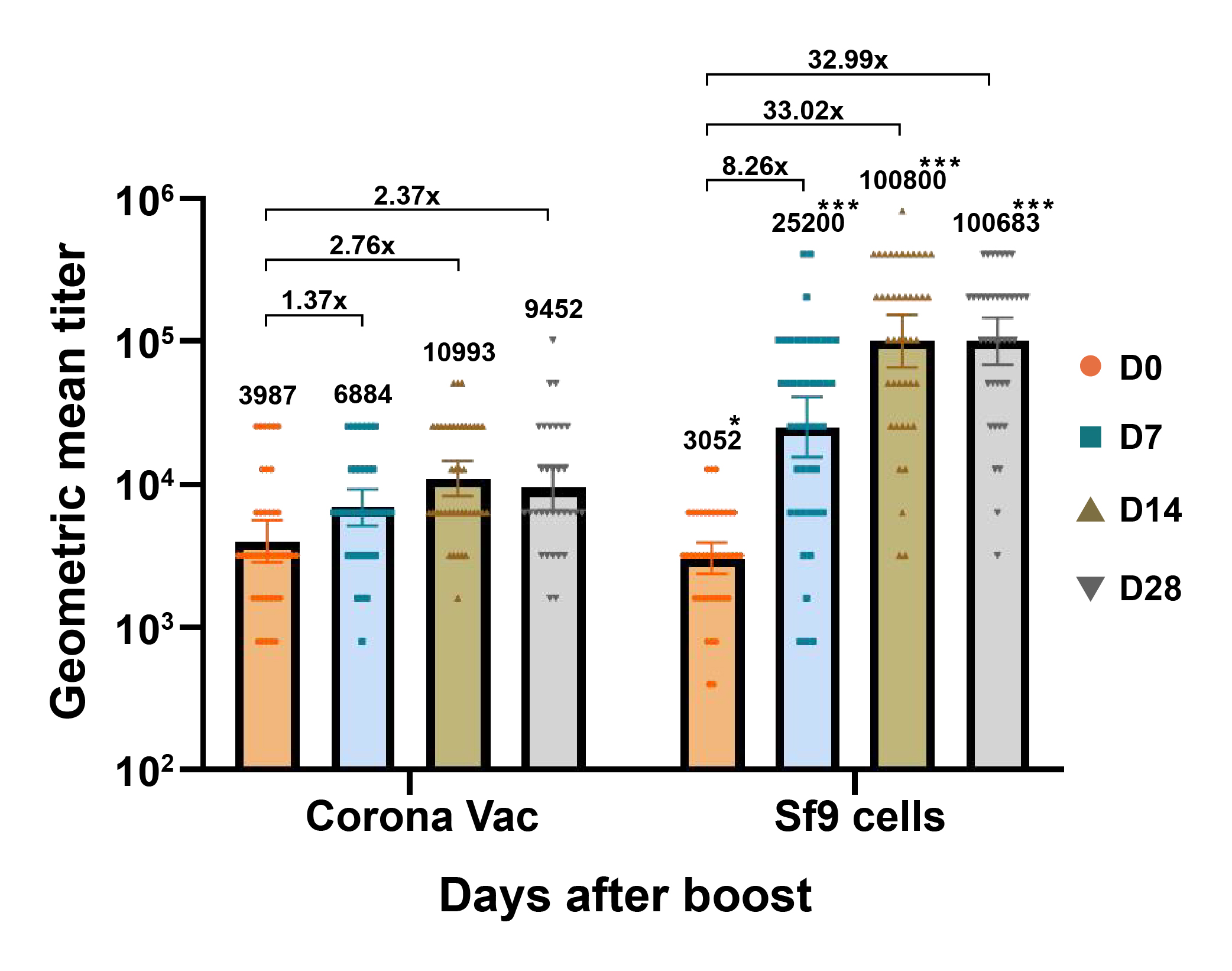

Supplement: Supplementary file 1 — Supplementary Figure 1 [file 41392_2024_1751_MOESM1_ESM.jpg]
