# Supplementary material for: Safety, immunogenicity and protective effectiveness of heterologous boost with a recombinant COVID-19 vaccine (Sf9 cells) in adult recipients of inactivated vaccines
Source: Signal Transduct Target Ther. 2024 Feb 14;9:41. doi: 10.1038/s41392-024-01751-1 (PMC10866951; doi:10.1038/s41392-024-01751-1)
Supplement: Supplementary file 2 — Supplementary Materials [file 41392_2024_1751_MOESM2_ESM.docx]

Supplementary Materials for

**Safety, immunogenicity and protective effectiveness of heterologous boost with a recombinant COVID-19 vaccine (Sf9 cells) in the inactivated vaccine adult recipients**

Wenxin Luo^1,2,3,4,5^, Jiadi Gan^1^, Zhu Luo^1,6^, Shuangqing Li^7,8^, Zhoufeng Wang^2,3,4^, Jiaxuan Wu^1^, Huohuo Zhang^1^, Jinghong Xian^1,2,3,4,9^, Ruixin Cheng^1^, Xiumei Tang^1,10^, Yi Liu^1^, Ling Yang^6^, Qianqian Mou^6,10^, Xue Zhang^6,10^, Yi Chen^11^, Weiwen Wang^12^, Yantong Wang^6^, Lin Bai^7^, Xuan Wei^7^, Rui Zhang^6^, Lan Yang^1,2,3,4^, Yaxin Chen^2^, Li Yang ^13^, Yalun Li^1,2,3,4^, Dan Liu^1,2,3,4,5^, Weimin Li^1,2,3,4,5,9^ and Lei Chen^14^

Correspondence to: Dan Liu (liudanscu@qq.com), Weimin Li (weimi003@scu.edu.cn), Lei Chen (leilei_25@126.com).

**This PDF file includes:**

Figure S1

Tables S1 to S5


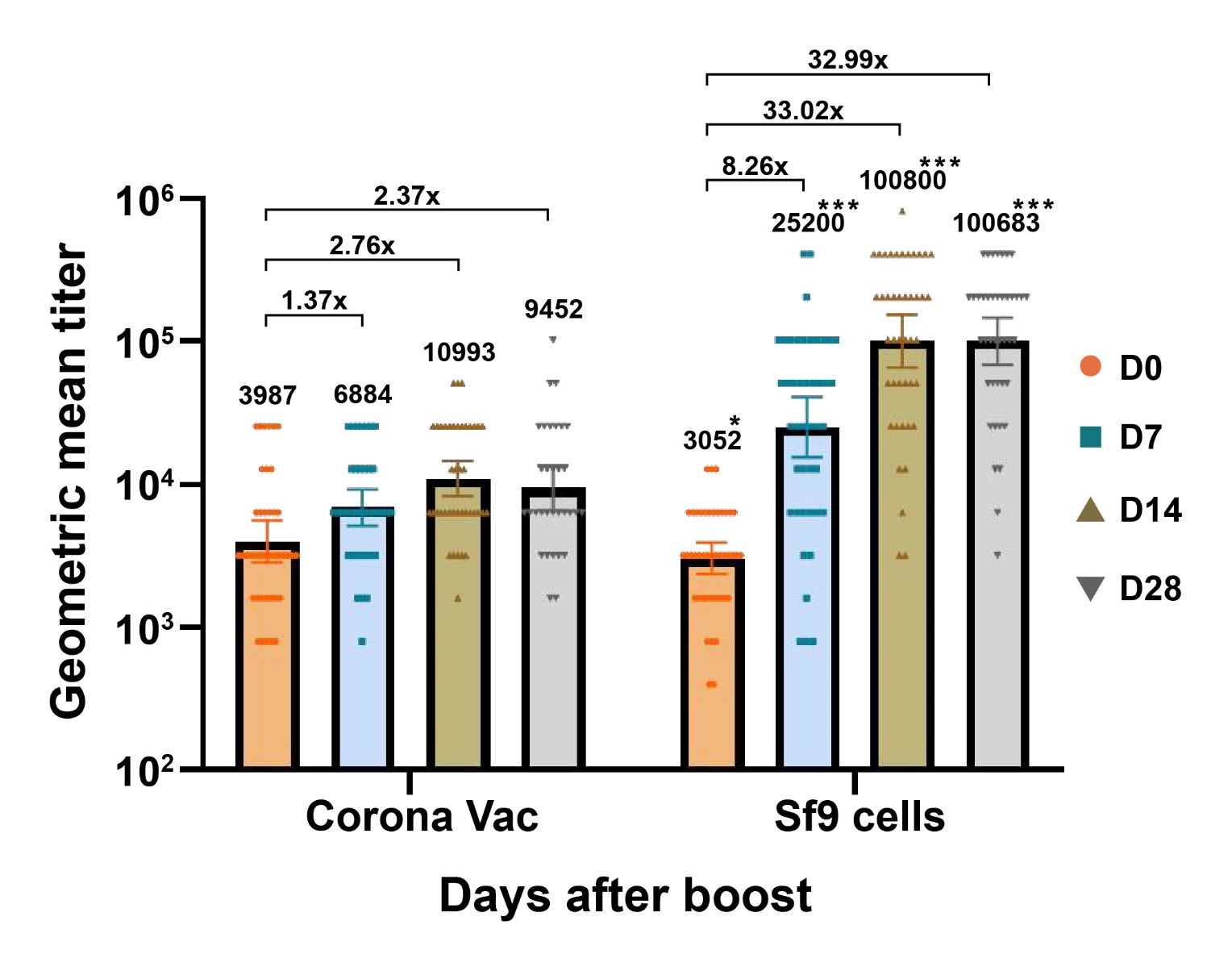


**Fig. S1 Geometric mean titers of binding antibodies against SARS-CoV-2 Prototype (HB-01) before and after the booster vaccination.** The numbers above each column indicate the median geometric mean titer, above which fold changes (e.g. "2.37×") are shown. **p* < 0.05, ***p* < 0.01, *** *p* < 0.001, **** *p* < 0.0001 (Unpaired two-sided Student’s *t* test). Abbreviation: CI, Confidence interval.

**Table S1. Binding antibody responses at day 0, 7, 14, and 28 after different types of boost vaccination**

| **Covariate** | **All**  **(n=85)** | **Heterologous boost with Sf9 cells vaccine**  **(n=44)** | **Homologous boost with CoronaVac vaccine**  **(n=41)** | ***p* value** |
| --- | --- | --- | --- | --- |
| **Binding antibodies to the RBD (GMT)** | | | | |
| Day 0 | 3471.88 (2823.07-4269.81) | 3052.29 (2366.06-3937.54) | 3986.56 (2846.18-5583.87) | 0.036 |
| Day 7 | 13811.79 (10018.54-19041.23) | 25199.87 (15528.41-40894.95) | 6884.42 (5131.20-9236.67) | <0.001 |
| Day 14 | 34615.97 (24425.55-49057.87) | 100799.50 (66053.54-153822.76) | 10993.35 (8282.00-14592.34) | <0.001 |
| Day 28 | 35691.88 (24423.45-52159.31) | 100683.37 (68820.24-147298.84) | 9451.69 (6565.94-13605.73) | <0.001 |

**Table S2. Neutralizing antibody responses at day 0, 7, 14, and 28 after different types of boost vaccination**

| **Covariate** | **All**  **(n=85)** | **Heterologous boost with Sf9 cells vaccine**  **(n=44)** | **Homologous boost with CoronaVac vaccine**  **(n=41)** | ***p* value** |
| --- | --- | --- | --- | --- |
| **Neutralizing antibodies to pseudo virus (GMT)** | | | | |
| Prototype (HB-01) | | | | |
| Day 0 | 121.99 (104.35-142.61) | 112.09 (85.14-147.57) | 132.77 (113.39-155.46) | 0.651 |
| Day 7 | 839.08 (680.82-1034.12) | 1785.27 (1547.64-2059.39) | 394.36 (320.71-484.94) | <0.001 |
| Day 14 | 1097.21 (857.27-1404.3) | 2628.47 (1991.91-3468.45) | 458.01 (400.92-523.24) | <0.001 |
| Day 28 | 1043.1 (852.35-1276.53) | 2490.09 (2340.48-2649.27) | 436.95 (399.71-477.66) | <0.001 |
| Delta (B.1.617.2) | | | | |
| Day 0 | 33.25 (28.56-38.71) | 33.99 (27.59-41.88) | 32.52 (25.82-40.96) | 0.812 |
| Day 7 | 434.07 (346.18-544.26) | 1099.89 (979.69-1234.84) | 171.30 (149.05-196.87) | <0.001 |
| Day 14 | 775.42 (627.15-958.74) | 1940.83 (1805.65-2086.12) | 309.80 (285.62-336.03) | <0.001 |
| Day 28 | 759.76 (614.13-939.92) | 1913.60 (1804.83-2028.93) | 301.65 (277.46-327.95) | <0.001 |
| Omicron BA.1 | | | | |
| Day 0 | 17.26 (14.37-20.74) | 14.72 (11.63-18.63) | 20.25 (15.26-26.87) | 0.020 |
| Day 7 | 263.83 (205.95-337.98) | 679.36 (609.76-756.9) | 102.46 (80.65-130.16) | <0.001 |
| Day 14 | 492.32 (390.21-621.14) | 1185.27 (956.56-1468.67) | 204.49 (178.75-233.94) | <0.001 |
| Day 28 | 494.04 (399.56-610.86) | 1138.72 (1005.77-1289.25) | 214.34 (182.00-252.43) | <0.001 |
| Omicron BA.2 | | | | |
| Day 0 | 16.72 (14.51-19.27) | 16.87 (13.49-21.11) | 16.57 (13.78-19.92) | 0.448 |
| Day 7 | 168.26 (134.93-209.82) | 409.36 (379.93-441.06) | 69.16 (57.76-82.81) | <0.001 |
| Day 14 | 325.52 (260.35-407.00) | 809.70 (696.26-941.62) | 130.86 (117.41-145.86) | <0.001 |
| Day 28 | 339.46 (271.81-423.94) | 823.93 (767.61-884.38) | 139.86 (115.49-169.37) | <0.001 |
| Omicron BA.2.75 | | | | |
| Day 0 | 23.41 (20.18-27.17) | 24.14 (19.85-29.36) | 22.70 (18.00-28.64) | 0.857 |
| Day 7 | 131.37 (106.01-162.81) | 326.15 (294.59-361.09) | 52.92 (48.20-58.09) | <0.001 |
| Day 14 | 274.36 (225.96-333.13) | 607.27 (536.71-687.10) | 123.95 (112.22-136.91) | <0.001 |
| Day 28 | 247.78 (198.18-309.79) | 605.56 (540.14-678.89) | 101.39 (85.74-119.88) | <0.001 |
| Omicron BA.3 | | | | |
| Day 0 | 7.50 (6.60-8.51) | 8.33 (6.79-10.23) | 6.75 (5.80-7.84) | 0.052 |
| Day 7 | 79.35 (63.36-99.37) | 200.21(170.88-234.57) | 31.45 (28.92-34.19) | <0.001 |
| Day 14 | 172.83 (137.08-217.89) | 420.95 (367.00-482.83) | 70.96 (58.16-86.56) | <0.001 |
| Day 28 | 153.67 (119.24-198.05) | 391.38 (362.58-422.47) | 60.34 (45.47-80.07) | <0.001 |
| Omicron BA.4/5 | | | | |
| Day 0 | 11.44 (9.90-13.22) | 12.25 (9.77-15.36) | 10.68 (8.85-12.89) | 0.168 |
| Day 7 | 85.63 (66.20-110.75) | 252.87 (219.50-291.32) | 28.99 (26.12-32.19) | <0.001 |
| Day 14 | 189.36 (152.20-235.60) | 466.78 (401.17-543.11) | 76.82 (71.11-82.99) | <0.001 |
| Day 28 | 180.80 (141.76-230.58) | 488.33 (420.09-567.66) | 66.94 (58.60-76.45) | <0.001 |
| Omicron BF.7 | | | | |
| Day 0 | 9.43 (8.05-11.05) | 12.84 (10.05-16.41) | 6.93 (5.92-8.12) | <0.001 |
| Day 7 | 72.17 (53.57-97.23) | 252.43 (212.07-300.45) | 20.63 (18.47-23.05) | <0.001 |
| Day 14 | 180.93 (134.39-243.60) | 451.59 (320.55-636.21) | 72.49 (54.92-95.69) | <0.001 |
| Day 28 | 171.59 (133.19-221.06) | 470.58 (392.87-563.67) | 62.57 (53.88-72.65) | <0.001 |
| **Neutralizing antibodies to live virus (GMT)** | | | | |
| Prototype (HB-01) | | | | |
| Day 0 | 31.72 (40.77-24.68) | 32.00 (22.28-45.96) | 31.45 (21.86-45.25) | 0.983 |
| Day 14 | 1630.68 (1987.24-1338.09) | 3628.10 (3331.45-3951.18) | 732.92 (631.58-850.52) | <0.001 |
| Omicron BA.1 | | | | |
| Day 0 | 10.56 (9.71-11.48) | 11.12 (9.83-12.57) | 10.02 (8.92-11.26) | 0.291 |
| Day 14 | 384.68 (324.13-456.54) | 749.61 (663.22-847.26) | 197.40 (175.13-222.51) | <0.001 |
| Omicron BA.2 | | | | |
| Day 0 | 9.68 (10.46-8.96) | 10.02 (8.92-11.26) | 9.35 (8.41-10.40) | 0.462 |
| Day 14 | 109.33 (127.97-93.41) | 1176.27 (1075.24-1286.79) | 177.32 (154.84-203.05) | <0.001 |
| Omicron BA.4 | | | | |
| Day 0 | 2.60 (2.97-2.27) | 2.51 (2.00-3.15) | 2.64 (2.22-3.14) | 0.634 |
| Day 14 | 256.00 (313.43-209.09) | 578.03 (517.31-645.88) | 113.38 (98.47-130.54) | <0.001 |
| Omicron BA.5 | | | | |
| Day 0 | 2.64 (3.01-2.31) | 2.64 (2.12-3.29) | 2.64 (2.22-3.14) | 0.706 |
| Day 14 | 186.88 (237.57-147.01) | 486.06 (428.15-551.81) | 71.85 (59.90-86.19) | <0.001 |

**Table S3. Comparison of the protective effectiveness against symptomatic COVID-19 in different types of boost vaccination**

| **Covariate** | **All**  **(n=85)** | **Heterologous boost with Sf9 cells vaccine**  **(n=44)** | **Homologous boost with CoronaVac vaccine**  **(n=41)** | ***p* value** |
| --- | --- | --- | --- | --- |
| **Symptomatic COVID-19 in entire cohort, n (%)** |  |  |  |  |
| Overall | 40 (47.06) | 14 (31.82) | 26 (63.41) | 0.004 |
| Inpatient or severe | 0 (0) | 0 (0) | 0 (0) | — |
| **Symptomatic COVID-19 in age groups, n (%)** |  |  |  |  |
| 18-59 years | 37 (43.53) | 12 (27.27) | 25 (60.98) | 0.002 |
| ≥60 years | 3 (3.53) | 2 (4.55) | 1 (2.40) | ＞0.999 |
| **Number of COVID-19 symptoms, n (%)** |  |  |  |  |
| ＜3 | 27 (31.76) | 11 (25.00) | 16 (39.02) | 0.165 |
| ≥3 | 13 (15.30) | 3 (6.82) | 10 (24.39) | 0.025 |

**Table S4. The inclusion and exclusion criteria**

| **Eligibility Criteria** |
| --- |
| －Healthy population aged 18 years or older, who voluntarily provide Informed Consent Forms (ICF) approved by the Ethics Committee prior to any research procedures, and agree to participate in this study. |
| －Subjects with medical history examination, physical examination, and clinical assessment confirming their health status, meeting the criteria for immunization with the investigational product. |
| －Subjects who have completed the vaccination procedure of the domestically approved inactivated vaccine with 3 doses administered more than 6 months prior to participating in this clinical trial (counting from the last vaccination date as day 0), and can provide relevant vaccination certificates. |
| －Subjects who are able and willing to adhere to the requirements of the clinical trial protocol and can complete the follow-up. |
| －Fertile males and females of childbearing potential voluntarily use effective contraception methods (such as condoms, intrauterine devices, spermicidal agents) from the time of signing informed consent until 6 months after vaccination. The use of oral contraceptives is not permitted. Female subjects with negative pregnancy tests agree not to breastfeed during the study period and at least 3 months after vaccination. |
| －Axillary temperature <37.3°C. |
| **Exclusion Criteria** |
| －Positive SARS-CoV-2 RT-PCR test result at screening. |
| －History of infection or disease related to severe acute respiratory syndrome coronavirus 2 (SARSCoV-2), severe acute respiratory syndrome (SARS), Middle East respiratory syndrome (MERS), etc. |
| －History of seizures, epilepsy, cerebral disease, or mental illness, either personally or within the family. |
| －Acupuncture syncope. |
| －Intending to become pregnant puerperal, or nursing or participate in sperm or egg donation during the trial period. |
| －History of hypersensitivity or allergic reactions to any vaccine or its components, such as allergies, urticaria, severe skin eczema, breathing difficulties, laryngeal edema, vasomotor angioedema, etc. |
| －Vaccination with any vaccine within 30 days prior to this study or planning to receive any other vaccine during the study period, except for the investigational vaccine. |
| －Participation in any other interventional trials, devices, or drug studies within 30 days prior to screening, or current use of investigational drugs within 5 half-lives after the last administration of the study drug. |
| －Genetic bleeding tendencies or coagulation abnormalities, severe bleeding history, significant bleeding or bruising after intramuscular injection or venipuncture, or history of purpura. |
| －Known medical history or diagnosis of diseases affecting the immune system function, including cancer, congenital or acquired immune deficiencies (e.g., HIV infection), uncontrolled autoimmune diseases. |
| －Presence of severe or uncontrollable respiratory, cardiovascular, neurological, hematologic, lymphatic, hepatic, renal, metabolic, or musculoskeletal diseases that, according to the investigator's judgment, would affect the evaluation of study outcomes. |
| －Absence of a spleen or functional asplenia. |
| － Continuous use of immunosuppressive agents or other immunomodulating drugs (such as corticosteroids: prednisone or similar drugs) for ≥14 days within 6 months prior to vaccination with the investigational vaccine. Topical treatments (e.g., ointments, eye drops, inhalers, or nasal sprays) are allowed if they do not exceed the recommended doses in the package insert or show any signs of systemic exposure. |
| －Receipt of immunoglobulins and/or blood products within 3 months prior to vaccination with the investigational vaccine. |
| －Patients undergoing anti-tuberculosis treatment. |
| －Individuals judged by the investigator to be incompatible with the trial protocol due to medical, psychological, social, or other conditions, or those affecting the participant's ability to provide informed consent. |

**Table S5. The diagnosis Criteria for COVID-19 disease**

| Suspected Cases: Subjects meeting the following clinical symptoms or presenting with radiological features characteristic of COVID-19. |
| --- |
| Clinical symptoms (meeting either ① or ② is considered symptomatic): |
| ① Presence of two or more of the following symptoms (persisting for ≥2 days): fever (oral temperature ≥38°C or axillary temperature ≥37.7°C), sore throat, general weakness/fatigue, rhinitis, muscle pain, headache, anorexia/nausea/vomiting, diarrhea, altered mental status. |
| ② Presence of one or more of the following symptoms (persisting for ≥2 days): cough, loss of smell or taste, difficulty breathing. |
| COVID-19 associated imaging features: |
| Chest X-ray: indistinct shadows, often circular, distributed in the peripheral and lower lung areas. |
| Chest CT: multiple bilateral ground-glass opacities, often circular, distributed in the peripheral and lower lung areas. |
| Endpoint cases: After more than 15 days post-vaccination, subjects meeting any of the following criteria are considered endpoint cases. |
| 1. Subjects with clinical symptoms consistent with suspected cases (with or without imaging features) and confirmed by virology (SARS-CoV-2 PCR) test. |
| 2. Subjects with clinical symptoms consistent with suspected cases (with or without imaging features) and tested positive for novel coronavirus antigen during the disease course. |
| 3. Subjects with clinical symptoms consistent with suspected pathological cases (with or without imaging features) and having a history of residing or traveling in the COVID-19 pandemic regions during the outbreak, or having definite contact with COVID-19 patients. |
